# Supplementary material for: Child diarrhea in Cambodia: A descriptive analysis of temporal and geospatial trends and logistic regression-based examination of factors associated with diarrhea in children under five years
Source: PLoS One. 2025 Jan 23;20(1):e0316155. doi: 10.1371/journal.pone.0316155 (PMC11756758; doi:10.1371/journal.pone.0316155)
Supplement: S1 Table — (DOCX) [file pone.0316155.s001.docx]

**Supporting information**

**S1 Table.** Results of checking multicollinearity using Variance Inflation Factor

| **Variable** | **VIF** |
| --- | --- |
| Wealth quintile | 2.16 |
| Type of toilet facility | 2.11 |
| Mother age | 1.50 |
| Survey years | 1.47 |
| Mother education | 1.45 |
| Place of residence | 1.32 |
| Geographical region | 1.24 |
| Smoking | 1.10 |
| Maternal emyploment | 1.02 |
| Sex of child | 1.00 |
| Mean VIF | 1.42 |
