# Supplementary material for: Child diarrhea in Cambodia: A descriptive analysis of temporal and geospatial trends and logistic regression-based examination of factors associated with diarrhea in children under five years
Source: PLoS One. 2025 Jan 23;20(1):e0316155. doi: 10.1371/journal.pone.0316155 (PMC11756758; doi:10.1371/journal.pone.0316155)
Supplement: Attachment — Submitted filename: PLOS ONE -REVIWEVER`S COMMENTS-2.docx [file pone.0316155.s003.docx]

**Reviewer`s comments- 2**

**The Authors have addressed a very important topic of childhood Diarrhoea Cambodia, Diarrhoea being a major cause of Morbidity and mortality among U5s in LMICs**

**This is a well-written paper in the Abstract the authors provided the prevalence of Diarrhoea but didn’t indicate the total sample size of children analyzed. This should be done. The second sentence of the abstract is too long. It should be revised accordingly.**

**The results were fairly well presented but tables 1,2 and 3 are very busy and long some covering 3 pages! Is here away these can be reduced e.g. some of the information written in text form. The table, graph and chats in the appendix are not numbered not titled. This should be done. The authors discussed their results well and made appropriate conclusions’ limitations should be revised to make them clear and concise. Did their study have any strength. They only talked about the study limitations.**
