# Supplementary material for: Child diarrhea in Cambodia: A descriptive analysis of temporal and geospatial trends and logistic regression-based examination of factors associated with diarrhea in children under five years
Source: PLoS One. 2025 Jan 23;20(1):e0316155. doi: 10.1371/journal.pone.0316155 (PMC11756758; doi:10.1371/journal.pone.0316155)
Supplement: Attachment — Submitted filename: Response to ReviewersR1.docx [file pone.0316155.s004.docx]

**Authors’ point-by-point responses to the editor and reviewers**

PONE-D-24-17960

Child diarrhea in Cambodia: A descriptive analysis of temporal and geospatial trends and logistic regression-based examination of factors associated with diarrhea in children under five years

PLOS ONE

Dear Dr. Um,

Thank you for submitting your manuscript to PLOS ONE. After careful consideration, we feel that it has merit but does not fully meet PLOS ONE’s publication criteria as it currently stands. Therefore, we invite you to submit a revised version of the manuscript that addresses the points raised during the review process.

In the abstract, the authors write “…used multivariate logistic regression…”. I think they used multivariable logistic regression. There is a huge difference between “multivariable” and “multivariate”. Check it carefully.

Author's response: We have considered the recommendations of both the editor and reviewers, reviewed the literature and online discussions related to naming conventions for these terms, and consulted with colleagues external to this study. We have opted to revise the use of multivariate to ***multiple logistic regression*** with reported adjusted odds ratio (AOR) throughout the manuscript.

In logistic regression, the authors use pooled data. Justify the use of combined data.

Author's response: We have revised as suggested.

I think, in this circumstance, spatiotemporal modeling is more effective than applying logistic regression in combined data.

Author's response:

A. We have revised the manuscript, both in the abstract and in the main text, to indicate that this analysis has two primary aims. The first aim is to **describe** temporal and geographic trends of diarrhea in children under five years old, as noted by the editor and reviewer. The second is to identify factors associated with diarrhea among children under five years old in Cambodia.

**Aim 1**: The editor and reviewer are correct that our results suggest minimal variation over time and that these variations are not statistically significant. While the differences across the temporal trend of diarrhea are not statistically significant, we believe that these results are substantively important — we believe it is important to document evidence that suggests little improvement in diarrhea among children under five years old during the time considered.

B. We concur with the reviewer that 4 points in time is less than ideal and have noted this as **a limitation in our discussion**. We likewise agree with the reviewer’s recommendations for a more sophisticated analytic approach and will look forward to engaging in that analysis once additional data become available. However, we believe that the current results are of scientific importance and worthy of documentation at the present time.

**Aim 2**: After describing the temporal and spatial trends in diarrhea, albeit with the limitations that we have acknowledged, we focus most of our analysis on examining ***factors associated*** with children under five years old in Cambodia. For this analysis, we did evaluate potential effect modification for all considered factors through the modeling of interaction terms with the temporal variable for year; however, the results from these analyses were not statistically significant. Given the lack of statistical significance of temporal differences, we were not surprised by the lack of effect modification with other factors. We have endeavored to revise the manuscript to delineate these observations more clearly in the discussion.

In Table 2, just keep the “% Diarrhea” column. No need to add “% Not Diarrhea”. It seems redundant because “% diarrhea + % Not diarrhea=100”.

Author's response: We reviewed and revised ***Table 2*** in the manuscript.

Please submit your revised manuscript by Nov 20 2024 11:59PM. If you will need more time than this to complete your revisions, please reply to this message or contact the journal office at plosone@plos.org. When you're ready to submit your revision, log on to https://www.editorialmanager.com/pone/ and select the 'Submissions Needing Revision' folder to locate your manuscript file.

Please include the following items when submitting your revised manuscript:

A rebuttal letter that responds to each point raised by the academic editor and reviewer(s). You should upload this letter as a separate file labeled 'Response to Reviewers'.

A marked-up copy of your manuscript that highlights changes made to the original version. You should upload this as a separate file labeled 'Revised Manuscript with Track Changes'.

An unmarked version of your revised paper without tracked changes. You should upload this as a separate file labeled 'Manuscript'.

If you would like to make changes to your financial disclosure, please include your updated statement in your cover letter. Guidelines for resubmitting your figure files are available below the reviewer comments at the end of this letter.

If applicable, we recommend that you deposit your laboratory protocols in protocols.io to enhance the reproducibility of your results. Protocols.io assigns your protocol its own identifier (DOI) so that it can be cited independently in the future. For instructions see: https://journals.plos.org/plosone/s/submission-guidelines#loc-laboratory-protocols. Additionally, PLOS ONE offers an option for publishing peer-reviewed Lab Protocol articles, which describe protocols hosted on protocols.io. Read more information on sharing protocols at https://plos.org/protocols?utm_medium=editorial-email&utm_source=authorletters&utm_campaign=protocols.

We look forward to receiving your revised manuscript.

Kind regards,

Md. Moyazzem Hossain

Academic Editor

PLOS ONE

Journal Requirements:

When submitting your revision, we need you to address these additional requirements.

1. Please ensure that your manuscript meets PLOS ONE's style requirements, including those for file naming. The PLOS ONE style templates can be found at

https://journals.plos.org/plosone/s/file?id=wjVg/PLOSOne_formatting_sample_main_body.pdf and

<https://journals.plos.org/plosone/s/file?id=ba62/PLOSOne_formatting_sample_title_authors_affiliations.pdf>

Author's response: We believe that we have followed these style requirements. We have used the PACE tool to revise any figures as needed. We do not want issues with formatting to constitute a barrier to publication, so if we have missed something, please let us know, we are more than willing to address any formatting errors that we may have missed.

2. Please provide additional details regarding participant consent. In the ethics statement in the Methods and online submission information, please ensure that you have specified what type you obtained (for instance, written or verbal, and if verbal, how it was documented and witnessed). If your study included minors, state whether you obtained consent from parents or guardians. If the need for consent was waived by the ethics committee, please include this information.

Once you have amended this/these statement(s) in the Methods section of the manuscript, please add the same text to the “Ethics Statement” field of the submission form (via “Edit Submission”).

For additional information about PLOS ONE ethical requirements for human subjects research, please refer to <http://journals.plos.org/plosone/s/submission-guidelines#loc-human-subjects-research>.

Author's response: The study participants were informed about the survey objectives before their interview, and **written** informed consent was obtained from their parents or guardians before data collection for each participant under 18 years old. The details about the final CDHS 2021-2022 report at (URL: <https://www.dhsprogram.com/publications/publication-FR377-DHS-Final-Reports.cfm>)

3. We note that [Figure 4] in your submission contain [map/satellite] images which may be copyrighted. All PLOS content is published under the Creative Commons Attribution License (CC BY 4.0), which means that the manuscript, images, and Supporting Information files will be freely available online, and any third party is permitted to access, download, copy, distribute, and use these materials in any way, even commercially, with proper attribution. For these reasons, we cannot publish previously copyrighted maps or satellite images created using proprietary data, such as Google software (Google Maps, Street View, and Earth). For more information, see our copyright guidelines: <http://journals.plos.org/plosone/s/licenses-and-copyright>.

Author's response: To clarify, the Cambodian provincial shapefile used in our figures was sourced directly from the United Nations Office for the Coordination of Humanitarian Affairs (OCHA), accessible at <https://data.humdata.org/dataset/cod-ab-khm>. We have confirmed that this dataset is compatible with a CC BY 4.0 license and is publicly available for research use.

We require you to either (1) present written permission from the copyright holder to publish these figures specifically under the CC BY 4.0 license, or (2) remove the figures from your submission:

a. You may seek permission from the original copyright holder of Figure 4 to publish the content specifically under the CC BY 4.0 license.

We recommend that you contact the original copyright holder with the Content Permission Form (http://journals.plos.org/plosone/s/file?id=7c09/content-permission-form.pdf) and the following text:

“I request permission for the open-access journal PLOS ONE to publish XXX under the Creative Commons Attribution License (CCAL) CC BY 4.0 (http://creativecommons.org/licenses/by/4.0/). Please be aware that this license allows unrestricted use and distribution, even commercially, by third parties. Please reply and provide explicit written permission to publish XXX under a CC BY license and complete the attached form.”

Please upload the completed Content Permission Form or other proof of granted permissions as an "Other" file with your submission.

In the figure caption of the copyrighted figure, please include the following text: “Reprinted from [ref] under a CC BY license, with permission from [name of publisher], original copyright [original copyright year].”

b. If you are unable to obtain permission from the original copyright holder to publish these figures under the CC BY 4.0 license or if the copyright holder’s requirements are incompatible with the CC BY 4.0 license, please either i) remove the figure or ii) supply a replacement figure that complies with the CC BY 4.0 license. Please check copyright information on all replacement figures and update the figure caption with source information. If applicable, please specify in the figure caption text when a figure is similar but not identical to the original image and is therefore for illustrative purposes only.

The following resources for replacing copyrighted map figures may be helpful:

USGS National Map Viewer (public domain): http://viewer.nationalmap.gov/viewer/

The Gateway to Astronaut Photography of Earth (public domain): http://eol.jsc.nasa.gov/sseop/clickmap/

Maps at the CIA (public domain): https://www.cia.gov/library/publications/the-world-factbook/index.html and https://www.cia.gov/library/publications/cia-maps-publications/index.html

NASA Earth Observatory (public domain): http://earthobservatory.nasa.gov/

Landsat: http://landsat.visibleearth.nasa.gov/

USGS EROS (Earth Resources Observatory and Science (EROS) Center) (public domain): http://eros.usgs.gov/#

Natural Earth (public domain): http://www.naturalearthdata.com/

[Note: HTML markup is below. Please do not edit.]

Reviewers' comments:

Reviewer's Responses to Questions

Comments to the Author

1. Is the manuscript technically sound, and do the data support the conclusions?

The manuscript must describe a technically sound piece of scientific research with data that supports the conclusions. Experiments must have been conducted rigorously, with appropriate controls, replication, and sample sizes. The conclusions must be drawn appropriately based on the data presented.

Reviewer #1: Partly

Reviewer #2: Yes

Author's Response: Revised according to recommendations provided by the reviewer #1

2. Has the statistical analysis been performed appropriately and rigorously?

Reviewer #1: Yes

Reviewer #2: Yes

3. Have the authors made all data underlying the findings in their manuscript fully available?

The PLOS Data policy requires authors to make all data underlying the findings described in their manuscript fully available without restriction, with rare exception (please refer to the Data Availability Statement in the manuscript PDF file). The data should be provided as part of the manuscript or its supporting information, or deposited to a public repository. For example, in addition to summary statistics, the data points behind means, medians and variance measures should be available. If there are restrictions on publicly sharing data—e.g. participant privacy or use of data from a third party—those must be specified.

Reviewer #1: Yes

Reviewer #2: Yes

4. Is the manuscript presented in an intelligible fashion and written in standard English?

PLOS ONE does not copyedit accepted manuscripts, so the language in submitted articles must be clear, correct, and unambiguous. Any typographical or grammatical errors should be corrected at revision, so please note any specific errors here.

Reviewer #1: No

Reviewer #2: Yes

Author's Response: Revised according to recommendations provided by the reviewer #1

5. Review Comments to the Author

Please use the space provided to explain your answers to the questions above. You may also include additional comments for the author, including concerns about dual publication, research ethics, or publication ethics. (Please upload your review as an attachment if it exceeds 20,000 characters)

Reviewer #1: 1- Mismatch between title and results: Authors put the title “Child diarrhea in Cambodia: A descriptive analysis of temporal and geospatial trends and logistic regression-based examination of factors associated with diarrhea in children under five years”, but the results partially match with the title. The results focus primarily on temporal trends in diarrhea prevalence, but did not examine the geographic variations of diarrhea as state in the title. Authors should revise the title accordingly.

Author's Response:

A. Again, we have revised the manuscript, both in the abstract and in the main text, to indicate that this analysis has two primary aims. The first aim is to **describe** temporal and geographic trends of diarrhea in children under five years old, as noted by the editor and reviewer. The second is to identify factors associated with diarrhea among children under five years old in Cambodia.

**Aim 1**: The editor and reviewer are correct that our results suggest minimal variation over time and that these variations are not statistically significant. While the differences across the temporal trend of diarrhea are not statistically significant, we believe that these results are substantively important — we believe it is important to document evidence that suggests little improvement in diarrhea among children under five years old during the time considered.

B. We concur with the reviewer that 4 points in time is less than ideal and have noted this as **a limitation in our discussion**. We likewise agree with the reviewer’s recommendations for a more sophisticated analytic approach and will look forward to engaging in that analysis once additional data become available. However, we believe that the current results are of scientific importance and worthy of documentation at the present time.

**Aim 2**: After describing the temporal and spatial trends in diarrhea, albeit with the limitations that we have acknowledged, we focus most of our analysis on examining ***factors associated*** with children under five years old in Cambodia. For this analysis, we did evaluate potential effect modification for all considered factors through the modeling of interaction terms with the temporal variable for year; however, the results from these analyses were not statistically significant. Given the lack of statistical significance of temporal differences, we were not surprised by the lack of effect modification with other factors. We have endeavored to revise the manuscript to delineate these observations more clearly in the discussion.

2- English writing: Authors should thoroughly review their English throughout the manuscript. For instance, from line 17 to 20 “The highest prevalence …… 2014 and 2022”, there is no verb.

Author's Response: Revised according to recommendations provided by the reviewer #1

3- Line 20-27: The presence of both odds ratios (ORs) less than 1 and greater than 1 indicates a relationship between the studied factors and childhood diarrhea. To avoid confusion, a more nuanced interpretation is necessary. Simply stating factors were 'independently associated' with diarrhea, like 'mothers aged 25-34 years (adjusted odds ratio (AOR) = 0.68; 95% CI: 0.48–0.96)', doesn't clarify tell the audiences whether this association is protective (OR < 1) or increases the risk (OR > 1) of diarrhea. Authors should provide a clearer explanation of the direction of the associations.

Author's Response: Revised according to recommendations provided by the reviewer #1

4- Line 27-29: Recommendation is too broad. Authors should provide more precise recommendation based on the findings.

Author's Response: We acknowledge the feedback regarding the recommendation being broad. Due to the word limit constraints of the abstract format, we were unable to provide more precise recommendations. However, we have included detailed, specific recommendations based on our findings in the main text of the manuscript.

5- Line 51-58: The manuscript focuses primarily on diarrhea. However, paragraph from line 51-58 are about under-five mortality which is a broader topic. It would be beneficial if the authors could address the specific contribution of diarrhea to overall under-five mortality within the paragraph. If data on this attribution is unavailable, it's best to omit this section entirely.

Author's Response: We appreciate the reviewer’s suggestion. We have revised the paragraph to specifically address the contribution of diarrhea to overall under-five mortality.

6- Line 134: Please omit “,” after “Plains included”

Author's Response: Revised accordingly in the manuscript's main text

7- Line 133-138: it would be easy to understand if authors puts the 5 geographical regions in a table.

Revised Response: We appreciate the reviewer’s suggestion. To enhance clarity, we have created a table to present the five geographical regions mentioned in lines 133-138, making it easier for readers to understand the information.

8- Line 147: “…. variations in the prevalence of anemia over time……” What does it mean?

Revised Response: We have revised the manuscript's main text accordingly. The phrase ‘variations in the prevalence of anemia over time’ has been updated to ‘variations in the prevalence of ***diarrhea*** over time’ to accurately reflect the outcome of interest.”

9- Table 1: “Had diarrhea in the past two weeks”. I assume that this refers to children. If so, please specify. To be easily understand the table, authors may capitalize main titles: “Had diarrhea in the past two weeks”, “Maternal Characteristics”, “Child Characteristics”, and “Household Characteristics”

Revised Response: We have revised **Table 1** accordingly.

10- Line 286-288: Please rewrite the sentence

Revised Response: Revised accordingly in the manuscript's main text

11- From Discussion part afterward, it is difficult to provide feedback to a specific part as there are no line numbers. Sometime, authors use the word “male and female” which are not appropriate for this age group.

Revised Response: We appreciate the reviewer’s feedback regarding the lack of line numbers in the Discussion section, which makes it challenging to provide specific feedback. We will consider including line numbers in the revised manuscript for easier reference. Additionally, we acknowledge the concern regarding the use of the terms ‘male and female’ in this age group. To be consistent with their age now, they're read as **boys** and **girls** in the manuscript.

12- In conclusion part: Authors should highlight factors associated with diarrhea across the survey years and/or geographical regions to reflex the title and research finding, rather than just mentioning x, y, … factors were associated with diarrhea.

Revised Response: Revised accordingly in the manuscript's main text in conclusion

Reviewer #2: The Authors have addressed a very important topic of childhood Diarrhoea Cambodia, Diarrhoea being a major cause of Morbidity and mortality among U5s in LMICs

This is a well-written paper in the Abstract the authors provided the prevalence of Diarrhoea but didn’t indicate the total sample size of children analyzed. This should be done. The second sentence of the abstract is too long. It should be revised accordingly.

Revised Response: Revised accordingly in the manuscript's main text in abstract

The results were fairly well presented but tables 1,2 and 3 are very busy and long some covering 3 pages! Is here away these can be reduced e.g. some of the information written in text form. The table, graph and chats in the appendix are not numbered not titled. This should be done. The authors discussed their results well and made appropriate conclusions’ limitations should be revised to make them clear and concise. Did their study have any strength. They only talked about the study limitations.

Revised Response: Revised accordingly in the manuscript's main text in strengths and limitations of the study

6. PLOS authors have the option to publish the peer review history of their article (what does this mean?). If published, this will include your full peer review and any attached files.

If you choose “no”, your identity will remain anonymous but your review may still be made public.

Do you want your identity to be public for this peer review? For information about this choice, including consent withdrawal, please see our Privacy Policy.

Reviewer #1: No

Reviewer #2: Yes: Prof. Edison Arwanire Mworozi
